# Factors Promoting Conduction Slowing as Substrates for Block and Reentry in Infarcted Hearts

**DOI:** 10.1016/j.bpj.2019.08.008

**Published:** 2019-08-12

**Authors:** Fernando O. Campos, John Whitaker, Radhouene Neji, Sébastien Roujol, Mark O’Neill, Gernot Plank, Martin J. Bishop

**Affiliations:** 1 School of Biomedical Engineering and Imaging Sciences, King’s College London, London, United Kingdom; 2 Institute of Biophysics, Medical University of Graz, Graz, Austria

## Abstract

The development of effective and safe therapies for scar-related ventricular tachycardias requires a detailed understanding of the mechanisms underlying the conduction block that initiates electrical re-entries associated with these arrhythmias. Conduction block has been often associated with electrophysiological changes that prolong action potential duration (APD) within the border zone (BZ) of chronically infarcted hearts. However, experimental evidence suggests that remodeling processes promoting conduction slowing as opposed to APD prolongation mark the chronic phase. In this context, the substrate for the initial block at the mouth of an isthmus/diastolic channel leading to ventricular tachycardia is unclear. The goal of this study was to determine whether electrophysiological parameters associated with conduction slowing can cause block and re-entry in the BZ. In silico experiments were conducted on two-dimensional idealized infarct tissue as well as on a cohort of postinfarction porcine left ventricular models constructed from ex vivo magnetic resonance imaging scans. Functional conduction slowing in the BZ was modeled by reducing sodium current density, whereas structural conduction slowing was represented by decreasing tissue conductivity and including fibrosis. The arrhythmogenic potential of APD prolongation was also tested as a basis for comparison. Within all models, the combination of reduced sodium current with structural remodeling more often degenerated into re-entry and, if so, was more likely to be sustained for more cycles. Although re-entries were also detected in experiments with prolonged APD, they were often not sustained because of the subsequent block caused by long-lasting repolarization. Functional and structural conditions associated with slow conduction rather than APD prolongation form a potent substrate for arrhythmogenesis at the isthmus/BZ of chronically infarcted hearts. Reduced excitability led to block while slow conduction shortened the wavelength of propagation, facilitating the sustenance of re-entries. These findings provide important insights for models of patient-specific risk stratification and therapy planning.

## Significance

Understanding ventricular tachycardia substrates is of great importance to improve treatment and the prognosis of patients with myocardial infarction. Specifically, the nature of electrophysiological remodeling within the border zone surrounding the infarct core and how this may lead to the initial block that results in ventricular tachycardia in the chronic phase of the disease remains unclear. In this study, we use biophysically detailed simulations to investigate the arrhythmogenic potential of experimentally reported changes in action potential duration as well as slowed conduction due to sodium current reduction, infiltration of fibrosis, and gap junction remodeling in the border zone. We provide quantitative data to demonstrate that functional and structural remodeling associated with conduction slowing provide the substrate for both arrhythmia initiation and maintenance.

## Introduction

Ventricular tachycardias (VTs) are associated with an increased risk of sudden death in patients with myocardial infarction (MI) (1). Clinical scar-related VTs are characterized by continuous circulating electrical wavefronts that repetitively re-excite the ventricular myocardium around a re-entrant pathway, or diastolic isthmus, generated by the region of scar and surviving tissue (2, 3). In the chronic phase of MI, these diastolic isthmuses are comprised of a heterogeneous admixture of remodeled myocardium and patchy fibrosis (4). Such tissue is often termed the infarct border zone (BZ) because it is also found at peripheral scar boundaries and increased presence of which has been correlated strongly with arrhythmic risk (5, 6).

The classical prerequisite for the genesis of an electrical re-entry is the combination of unidirectional block and slowed conduction (7), with its subsequent maintenance depending on the wavelength of the electrical impulse (8), along with the anatomical pathlength around the scar substrate (2, 3). A re-entry can only be sustained if the wavelength, given by the mathematical product of the conduction velocity (CV) and the effective refractory period (closely related to the action potential duration (APD)), is shorter than the conducting pathway within the heart (9).

Electrophysiological alterations resulting from functional and structural remodeling processes secondary to MI are believed to play a key role in the arrhythmic substrate. For example, downregulation of repolarizing potassium currents that act to prolong APD has been reported in experimental measurements with isolated myocytes from the BZ of canine hearts 5 days after MI (10). APD prolongation can lead to the dispersion of repolarization between the BZ and the neighboring healthy myocardium, providing a known potent substrate for unidirectional conduction block (11). Indeed, computational studies from our group (12, 13, 14) and others (15, 16, 17, 18, 19, 20, 21, 22) have implemented repolarizing potassium channel remodeling to enforce prolonged APD in the BZ as a reliable means of facilitating unidirectional block around the scar. Such an in silico approach has been particularly valuable in helping to understand cardiac arrhythmic mechanisms and, more recently, move toward patient-specific simulation and therapy planning (23).

However, experimental data obtained at the chronic phase of the disease, the time point in which most medical images used to build the computational models are obtained, suggest that remodeling processes promoting CV slowing as opposed to APD prolongation dominate (5, 24). CV is influenced by several factors, such as fast sodium current (INa) density, gap junction conductance, and integrity of the cardiac extracellular matrix. Fibrosis, frequently reported in the infarcted heart, disrupts the extracellular matrix, producing a profound impact on electrical activation patterns and timings (25). Strands of surviving myocytes interspersed with patchy fibrosis provide a substrate for structurally constrained conduction pathways, causing local propagation delays that manifest as macroscopic conduction slowing (6, 25, 26). Moreover, decoupling of neighboring cells by fibrotic inlays can give rise to isthmuses within the infarct scar with regions of rapid tissue expansion from the BZ to the myocardium, in which source-sink mismatch is prominent (27) and which can by themselves facilitate unidirectional block (12, 28, 29). Asymmetries in source-sink mismatch in these structural expansions are also exacerbated by conditions that impair tissue excitability, such as reduced INa (30). Reduced excitability at the mouth of a slow conducting isthmus with normal APD could therefore precipitate unidirectional conduction block while at the same time facilitating re-entry (as wavelength is not prolonged), providing a potentially lethal arrhythmogenic substrate.

Gaining in-depth understanding of the specific mechanisms underlying block and re-entry at different stages of MI is essential for the successful development of therapeutic strategies for scar-related VTs. In this study, we conduct in silico experiments to dissect the role played by different functional and structural remodeling processes in VT initiation within the chronically infarcted heart. Specifically, we investigate whether conditions promoting conduction slowing rather than abnormal repolarization can form a substrate for VT initiation (conduction block) and sustenance (reduced wavelength).

Slow CV resulting from reduced INa, decreased tissue conductivity, and fibrosis in the infarct BZ were tested alone and combined to assess their arrhythmogenic potential. Idealized as well as high-resolution infarct left ventricular (LV) models based on ex vivo porcine magnetic resonance imaging (MRI) data are used here to gain a thorough understanding of how functional and structural conduction slowing can lead to block and re-entry. Our results provide important knowledge regarding VT induction in the postinfarction heart.

## Materials and Methods

### Geometrical models

All simulations in this work were carried out in geometrical finite element (FE) models of two-dimensional (2D) idealized infarct anatomies as well as in a cohort of seven postinfarction porcine LV models reconstructed from ex vivo contrast-enhanced MRI scans.

#### 2D idealized model

**Figure 1:**
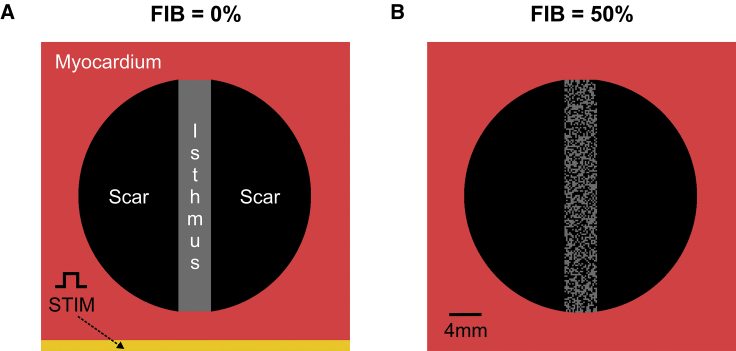
Schematic of the 2D computational model. (*A*) Shown is the myocardial tissue (*red*) with an idealized infarct scar (*black*) transcended by an isthmus of remodeled myocytes (*gray*). S1–S2 stimuli were applied to all cells in the lowermost portion of the tissue. (*B*) The same 2D model in (*A*) with 50% fibrosis (FIB) within the conducting isthmus is shown. To see this figure in color, go online.

A 4 × 4 cm cardiac sheet containing an idealized infarct region was meshed with a nodal spacing of 200 *μ*m resolution (40,000 quadrilateral FEs). As illustrated in Fig. 1 *A*, the infarct consisted of two semicircular segments representing a myocardial scar transcended by a 4 mm conducting isthmus. The scar was represented as being nonconducting (i.e., by imposing no-flux boundary condition at its interface).

#### High-resolution image-based LV models

##### Ex vivo MRI data

The high-resolution images used to build the computational models here were acquired in a different study from our group (unpublished data). In summary, in seven domestic pigs, the midleft anterior descending artery was occluded for 180 min to create experimental MI. The animals were euthanized 6 weeks post-MI using intravenous potassium chloride (KCl). Before euthanasia, the contrast-enhancement media gadolinium was administered to assess focal myocardial fibrosis. After excision, the hearts were filled with a three-dimensional (3D) scaffold printed in a flexible material (TangoPlus FullCure930 plastic) using an Objet500 Connex1, polyjet 3D printer (Objet-Stratasys, Rehovot, Israel). Hearts from all animals underwent ex vivo contrast-enhanced MRI suspended in a saline bath using a 1.5T scanner (MAGNETOM Aera; Siemens Healthcare, Erlangen, Germany). Images were obtained using an 0.4 × 0.4 × 0.4 mm^3^ isotropic 3D T1-weighted gradient echo sequence. A four-label structural segmentation of the LV blood pool (excluding papillary muscles), myocardium, infarct scar, and BZ was manually performed using the open source package Seg3D (www.seg3d.org). The infarct region was detected using a SI thresholding approach. First, a region of interest (ROI) in an enhanced portion of tissue that was within the vascular territory of the infarct was manually selected. Then, the mean and SD of the signal intensity (SI) within the ROI were calculated automatically in MATLAB (The MathWorks, Natick, MA). Next, the scar was defined as tissue with SI > mean (SI_*ROI*_) − SD (SI_*ROI*_). Finally, the BZ was defined as tissue with SI > mean (SI_*ROI*_) − 2 × SD (SI_*ROI*_) that was within 2 cm of the main body of the scar.

##### Mesh generation

**Figure 2:**
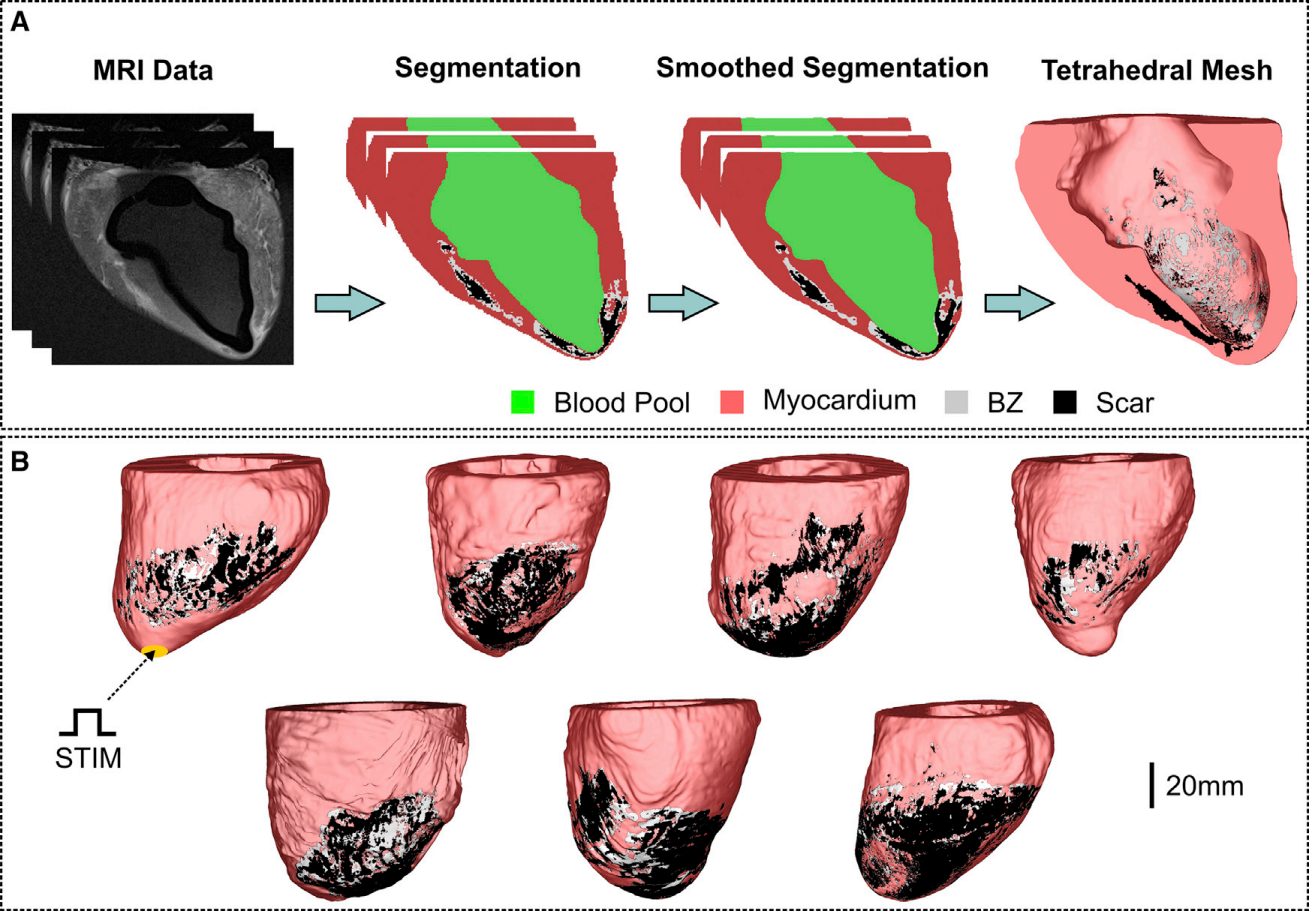
Generation of computational models of the porcine LV. (*A*) Shown is the workflow for the generation of high-resolution tetrahedral FE meshes of the LV from late gadolinium enhancement MRI stacks. Resulting image segmentation is as follows: blood pool (*green*), healthy myocardium (*red*), infarct BZ (*gray*), and scar (*black*). (*B*) Shown is the cohort of seven high-resolution 3D LV structure models. S1–S2 stimuli were applied to all cells in the apex of the LV. To see this figure in color, go online.

The pipeline to convert MRI data into 3D anatomic FE meshes is illustrated in Fig. 2. Before mesh generation, a smoothing and upsampling step was performed on the segmented images to increase their spatial resolution from the clinical (mm) to the modeling scale (*μ*m) (31). Tetrahedral FE meshes were created based on the smoothed segmentations using Tarantula (CAE Software Solutions, Eggenburg, Austria). The resulting LV models were comprised, on average, of 10,180,328 myocardial nodes defining 59,514,785 tetrahedral FEs with a mean discretization of 314 *μ*m (32). Myocardial fiber orientations were incorporated into the models using a rule-based approach (33).

### Modeling fibrosis in the BZ

Fibrosis was represented by including synthetic patterns of nonconducting tissue with different densities and random topologies into the isthmus/BZ of the geometrical models (27, 34) (see Fig. 1 *B*). Fibrosis was modeled here in the same way as the infarct scar (*i.e.*, by imposing no-flux boundary conditions along its interface). If not stated otherwise, the amount of fibrosis in relation to surviving myocardium (FIB) was varied from 0% (no fibrosis) to 90% in steps of 5%. 10 fibrotic patterns were constructed for each FIB to allow a statistical analysis to be taken. Fibrosis was added only into one of the LV models due to computational restrictions.

### Biophysical simulations

#### Model of the ventricular action potential

The ten Tusscher (TT) model (35) was used to simulate ionic membrane dynamics in endocardial myocytes from the porcine ventricles. Following previous computational studies (12, 13, 14, 15, 16, 17, 18, 19, 20, 21, 22), ionic cell properties in the tissue within the isthmus and infarct BZ were adjusted to produce:

1) Prolonged APD: the conductances of the rapid delayed rectifier potassium current and the slow delayed rectifier potassium current were reduced to 20 and 30% of their control values, respectively.
2) Reduced INa: the conductance of INa was adjusted according to a scaling factor (from 0.1 to 1.0, control, in steps of 0.05 if not stated otherwise). This allowed us to explore intersubject variations as well as to probe the effects of more extreme cases not accounted for in most previous studies, in which the conductance of INa was reduced to 38%.

#### Model of the electrical activity in cardiac tissue

Cardiac electrophysiology was simulated within all geometrical models using the monodomain formulation:

(1) $$\nabla \cdot \left(\sigma_{m}\nabla V_{m}\right)=\beta I_{m},$$

(2) $$C_{m}\frac{\partial V_{m}}{\partial t}+I_{ion}\left(V_{m},\eta \right)-I_{stim}=I_{m},$$

(3) $$\frac{\partial \eta }{\partial t}=f\left(V_{m},\eta \right),$$

where *σ*_*m*_ = diag (*σ*_*ml*_, *σ*_*mt*_, *σ*_*mt*_) is the harmonic mean conductivity tensor (36); *V*_*m*_ is the transmembrane voltage; *β* = 0.14 *μ*m^−1^ is the surface to volume ratio; *I*_*m*_ is the transmembrane current density; *C*_*m*_ is the membrane capacitance per unit area; *I*_*ion*_ is the density of the total ionic current flowing through the membrane channels, pumps, and exchangers as described in the TT model (35); and *I*_*stim*_ is the stimulus current density. *I*_*ion*_ depends on *V*_*m*_ as well as on *η*, a vector of state variables describing channel gating and ionic concentrations.

In the 2D sheets, isotropic conductivity was assigned to the tissue with a value of *σ*_*ml*_ = *σ*_*mt*_ = 0.143 S/m to produce a longitudinal CV of 0.6 m/s (37). Anisotropic conductivities values of *σ*_*ml*_ = 0.143 S/m and *σ*_*mt*_ = 0.064 S/m were assigned to the myocardium of the LV models, such that longitudinal and transverse CVs (*v*_*l*_ = 0.6 m/s, *v*_*t*_ = 0.4 m/s) fell within the range of velocities recorded in the ventricles (37). The BZ of the LV models was set to be isotropic with conductivity values of *σ*_*ml*_ = *σ*_*mt*_ = 0.064 S/m to account for differences in anisotropy between the healthy myocardium and the BZ (38). Moreover, *σ*_*m*_ in the BZ of the computational models was adjusted by a scaling factor (from 0.1 to 1.0, control, in steps of 0.05 if not stated otherwise) to gauge the effects of mild and severe reduction in tissue conductivity due to gap junction uncoupling and fiber disarray.

#### Pacing protocol

The Cardiac Arrhythmia Research Package (39) (http://carp.meduni-graz.at) was employed to simulate the electrical activity within the computational models in this study. Tissue- and organ-scale models were initialized with stabilized single-cell model states. Stabilized states were obtained after pacing the TT model at 2.0 Hz for 100 cycles. Propagation was initiated by applying a stimulus *I*_*stim*_ of 100 *μ*A/cm^2^ for 1 ms according to the S1–S2 protocol. Three S1 beats at a basic cycle length of 500 ms followed by a premature S2 with a shorter coupling interval were simulated. The time of the S2 beat was chosen by shortening the coupling interval from 500 ms in steps of 10 ms until propagation failure was detected. In the 2D idealized infarct model, *I*_*stim*_ was applied in the lowermost portion of the tissue (Fig. 1 *A*), whereas the LV models were paced at the apex (Fig. 2 *B*).

### Data analysis

The number of simulations in which conduction block, *n*, of the S2 beat was observed was recorded to compute the probability *P*_*B*_ = *n*/*N*, where *N* represents the number of instances (topologies) of fibrosis in the 2D model or the number of pigs in the cohort. Likewise, the probability of the blocked S2 beat to degenerate into re-entry was also determined. The re-entry vulnerability index (RVI) was used to detect the presence of conduction block and re-entry (11, 14). The RVI is a metric to quantify the likelihood of wavefront-waveback interactions around a re-entrant circuit (11). Re-entry initiation around a circuit requires that a wavefront traveling along a line of block finds tissue that has already regained excitability, enabling its reactivation. The RVI provides a point-by-point quantification of this principle by computing the time interval between the arrival of the wavefront at the exit site and the regaining of excitability (repolarization) of tissue just proximal to it (14). During normal propagation, RVI maps resemble the APD distribution in the tissue, whereas in the presence of block, absolute RVIs are smaller than the APD, becoming negative if a re-entry is induced. Here, regions with RVIs smaller than 30% of the average tissue APD were considered as areas of conduction block. Re-entries were visually checked and classified as sustained if they lasted for more than one cycle. Simulations in which the isthmus or BZ were completely inexcitable were not computed in *P*_*B*_.

## Results

The S1–S2 pacing protocol described above in Pacing protocol was used to investigate the occurrence of block and re-entry within the computational models in Figs. 1 and 2. In all tissue/organ-scale models, a premature S2 with a coupling interval of 320 ms was capable of inducing capture in the myocardium. Conduction slowing due to functional and structural remodeling in the BZ are assessed separately and combined in the following sections. Experiments with a 2D model with prolonged APD in the isthmus was carried out to serve as a basis for comparison.

### Arrhythmia induction in 2D sheets with idealized infarct scars

#### Induction due to prolonged APD

**Figure 3:**
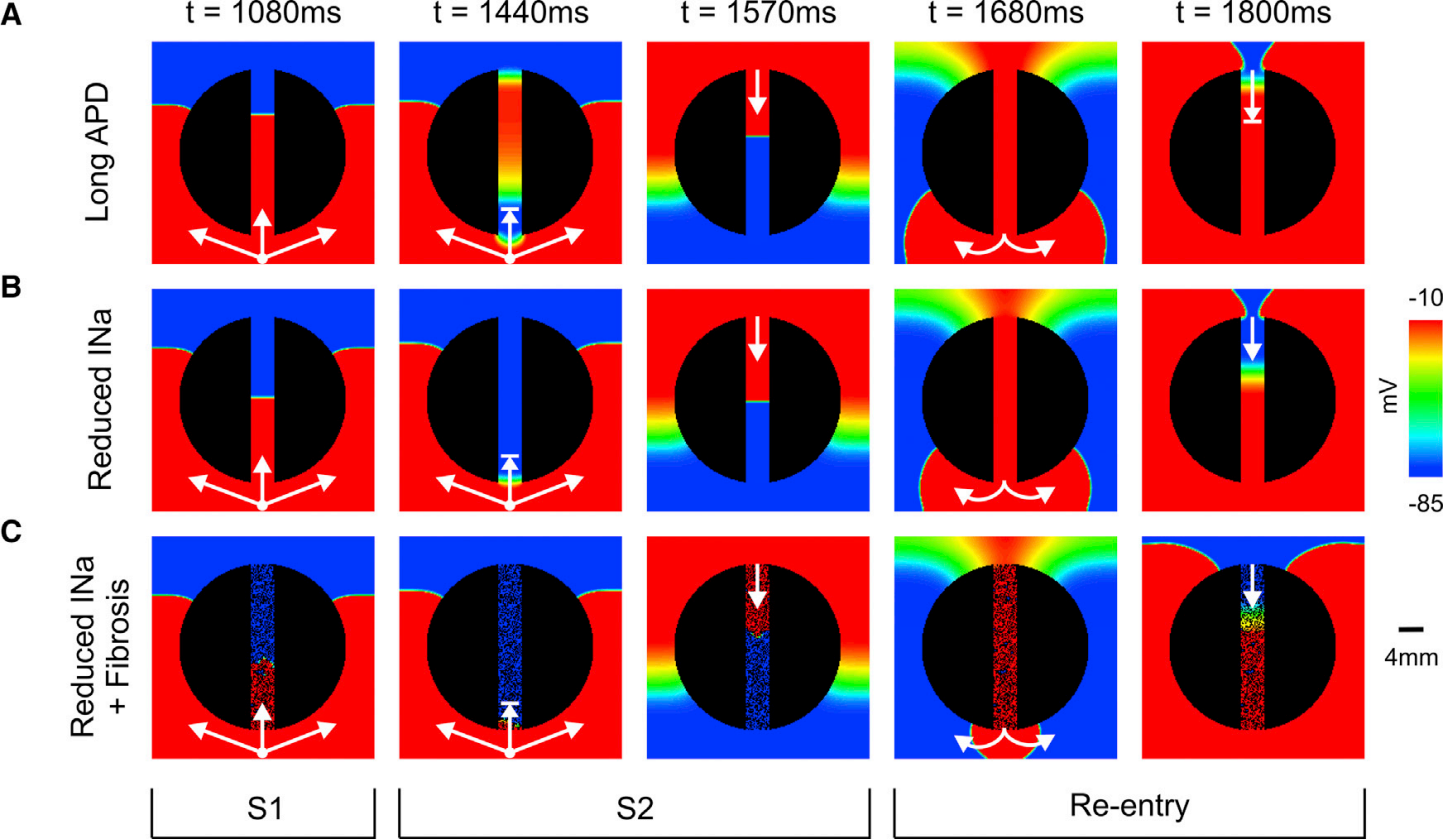
Arrhythmia within idealized infarct models with different remodeled isthmuses. *V*_*m*_ maps at different times show arrhythmia induction following the S1–S2 pacing protocol. (*A*) Nonsustained arrhythmia in a 2D sheet with prolonged APD in the isthmus is shown. (*B*) Sustained arrhythmia in a 2D sheet with reduced INa (30%) in the isthmus is shown. (*C*) Sustained arrhythmia in a 2D sheet with reduced INa (70%) and fibrosis (FIB = 50%) in the isthmus is shown. Arrows represent successful propagation. Lined arrows represent conduction block. Re-entry induction in the idealized infarct models shown in (*A*–*C*) can be fully appreciated in Videos S1, S2, and S3, respectively. To see this figure in color, go online.

Fig. 3 *A* shows the spatial distribution of *V*_*m*_ of the paced (last S1 and S2) and re-entrant wavefronts within a 2D idealized infarct model with prolonged APD in the isthmus. The S1 beat successfully propagates throughout the 2D sheet, whereas the S2 beat blocks at the isthmus’s mouth proximal to the stimulus site (t = 1440 ms). In this scenario, conduction block occurs because cells in the isthmus are still refractory upon the arrival of the premature S2 wavefront. The wavefront propagates around the nonconducting scar reaching the distal mouth of the isthmus, in which the tissue has had more time to regain excitability. At time t = 1570 ms, the wavefront propagates downwards through the isthmus re-entering the recovered myocardium at t = 1680 ms. Next, the re-entrant wavefront again travels around the scar but blocks at the distal mouth as the cells are still in the refractory period because of the prolonged APD. See Video S1 for further details of the S1, S2, and the nonsustained re-entrant wavefronts.

Video S1. Nonsustained Arrhythmia Induction within an Idealized Infarct Model with Prolonged APD in the Isthmus See Fig. 3 *A* for details. *V*_*m*_ of the last S1 beat (delivered at t = 1000 ms) followed by a premature S2 with a coupling interval of 320 ms that degenerated into a nonsustained re-entry (same scale as Fig. 3).

#### Induction due to reduced INa and structural remodeling

Fig. 3, *B* and *C* shows how reduced INa and fibrosis, factors promoting reduced excitability and disrupted conduction in infarcted tissue, can lead to block and re-entry. Decreased tissue conductivity itself was not capable to induce conduction block. Note that in both cases (Fig. 3, *B* and *C*), CV is slower than the scenario with prolonged APD (Fig. 3 *A*) during the S1 beat. Unlike in Fig. 3 *A*, the conduction block of the premature S2 beat at t = 1440 ms ensues because cells located at the proximal mouth have not had time to recover excitability due to the reduced INa. In both cases, the wavefront exiting from the proximal mouth (t = 1680 ms) can again re-enter the infarct region from the distal mouth as the isthmus has recovered excitability at t = 1800 ms. It can be seen, however, that CV is slower in the fibrotic isthmus because the wavefront is forced to travel through zig-zag pathways. Initiation and maintenance of both re-entries can be seen in Videos S2 and S3.

Video S2. Sustained Arrhythmia Induction within an Idealized Infarct Model with Reduced INa, 30%, in the Isthmus See Fig. 3 *B* for details. *V*_*m*_ of the last S1 beat (delivered at t = 1000 ms) followed by a premature S2 with a coupling interval of 320 ms that degenerated into a nonsustained re-entry (same scale as Fig. 3).

Video S3. Sustained Arrhythmia Induction within an Idealized Infarct Model with Reduced INa, 70%, and Fibrosis, FIB = 50%, in the Isthmus See Fig. 3 *C* for details. *V*_*m*_ of the last S1 beat (delivered at t = 1000 ms) followed by a premature S2 with a coupling interval of 320 ms that degenerated into a nonsustained re-entry (same scale as Fig. 3).

#### Arrhythmogenic potential of functional and structural conduction slowing

**Figure 4:**
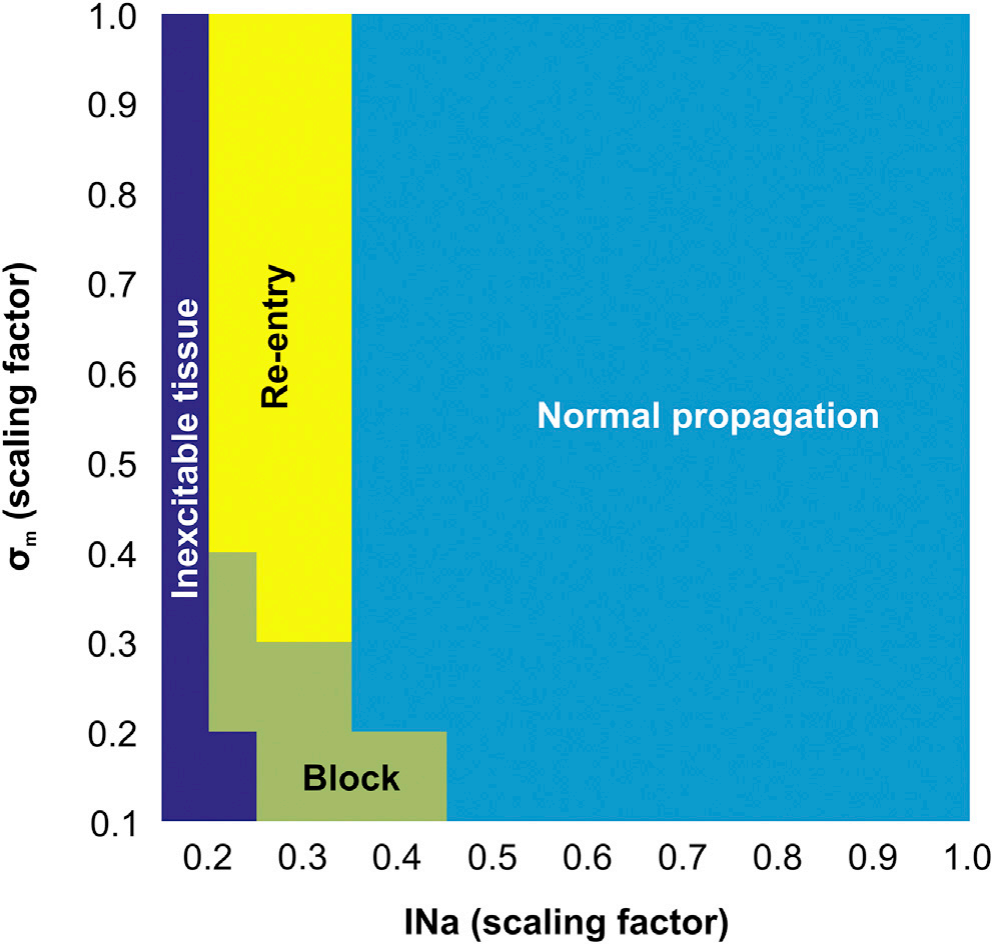
Parameter diagram showing the vulnerability of the 2D idealized infarct model to conduction block and re-entry as a function of *I*_*Na*_ and *σ*_*m*_. To see this figure in color, go online.

**Figure 5:**
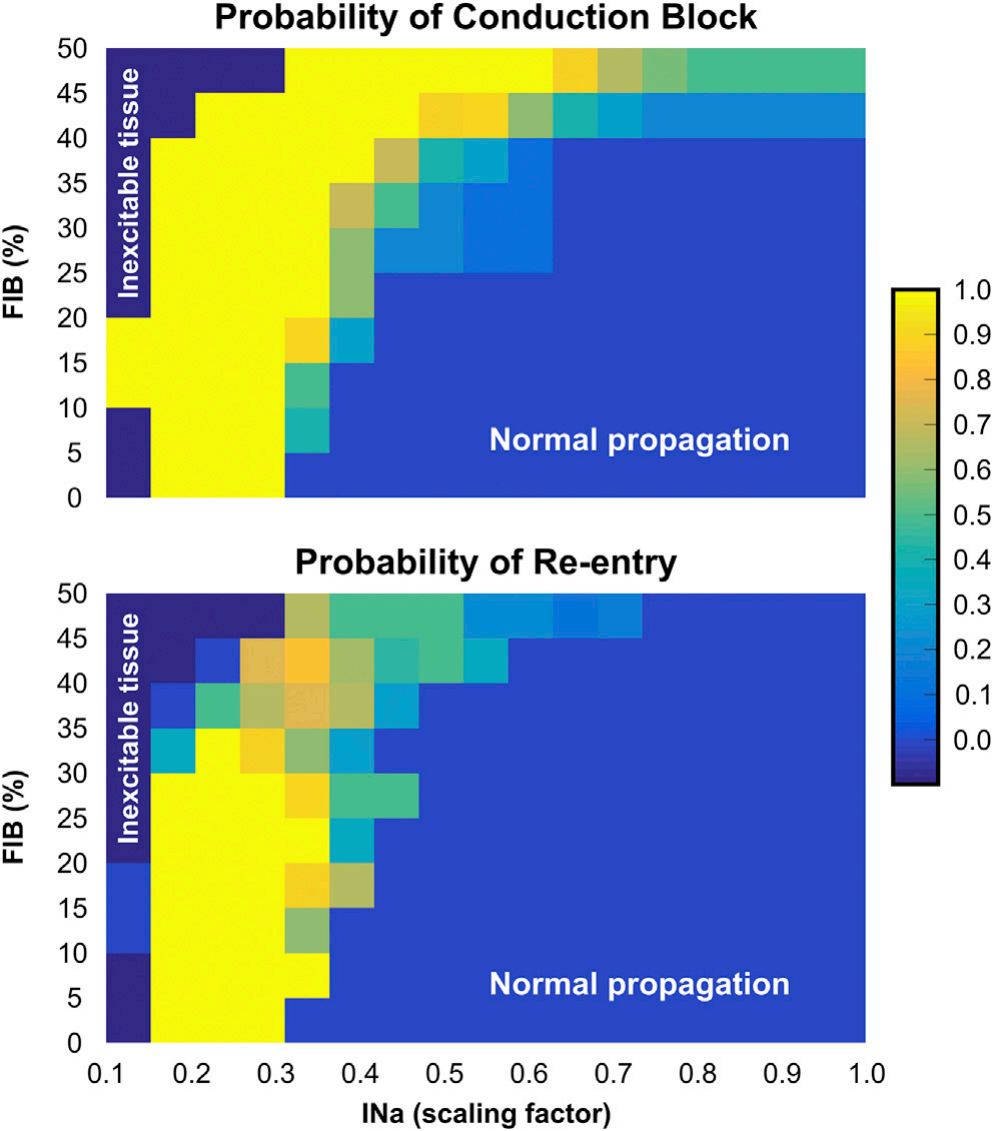
Heat map showing probabilities of conduction block and re-entry in the 2D idealized infarct model as functions of *I*_*Na*_ and FIB. To see this figure in color, go online.

**Figure 6:**
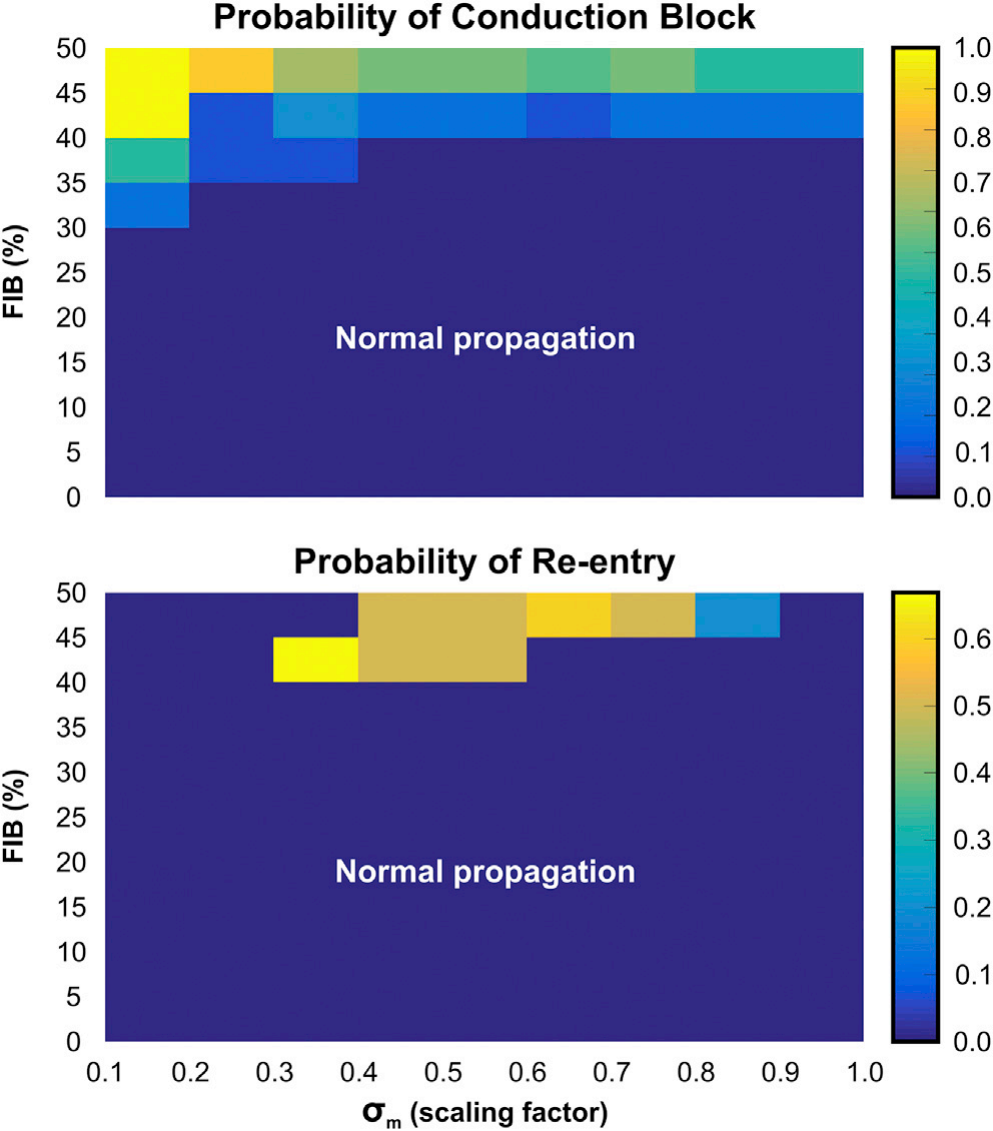
Heat map showing probabilities of conduction block and re-entry in the 2D idealized infarct model as functions of *σ*_*m*_ and FIB. To see this figure in color, go online.

The ability of factors associated with conduction slowing to promote or prevent arrhythmia induction in idealized infarct models are evaluated in Figs. 4, 5, and 6. In total, 2080 simulations were performed with different combinations of the three parameters (INa, *σ*_*m*_, and FIB) investigated in this study to compute statistics on the probability of block and re-entry.

Fig. 4 presents how reduced INa (functional remodeling) and decreased tissue conductivity mimicking gap junction uncoupling and fiber disarray (structural remodeling) can cause block and re-entry. In general, highly reduced INa resulted in the tissue becoming inexcitable. On the contrary, more normal INa led to normal propagation and, on the whole, an absence of block/re-entry. As mentioned above, reductions in *σ*_*m*_ alone were not capable to induce conduction block. However, decreased *σ*_*m*_ when combined with reduced INa could promote conduction block that may or may not degenerate into re-entry. In these cases, re-entry was more likely for lower INa combined with more preserved *σ*_*m*_.

Next, in Fig. 5, we analyzed how the combination of reduced INa and fibrosis may lead to arrhythmia in the idealized model. Unlike in decreased *σ*_*m*_ (Fig. 4), the presence of fibrosis itself led to the conduction block of the S2 beat. Block and re-entry were most likely to occur when INa was moderately reduced and in the presence of some degree of fibrosis. The smaller the reduction in INa, the larger the degree of fibrosis required to cause block/re-entry, and the larger the reduction in INa, the lower the required amount of fibrosis necessary. Neither the S2 nor S1 beats could propagate through isthmuses with FIB > 50% because the cells were completely disconnected from the healthy myocardium by fibrotic patches.

The effects of structural remodeling (*σ*_*m*_ and fibrosis) on arrhythmia induction are shown in Fig. 6. Decreased *σ*_*m*_ alone is not arrhythmogenic as there is no substrate for the initial block. However, when combined with (relatively high) levels of fibrosis, such a substrate is then present, and re-entry occurs. In these cases of high fibrosis, re-entry is more likely for lower *σ*_*m*_ because of the reduced wavelength.

### VT induction in MRI-based LV models

Functional and structural remodeling as substrates for block/re-entry were also investigated in the context of more complex scar anatomies. The analysis performed above on 2D idealized models was repeated on a cohort (n = 7) of MRI-based postinfarction LV models. However, the parameter search space (INa and *σ*_*m*_ scaling factors) explored in the 2D models had to be reduced because of the high computational cost associated with the LV models. Thus, both INa and *σ*_*m*_ were scaled by factors of 1.0 (control), 0.50, 0.25, and 0.10 in the BZ of all models in the cohort. As in the 2D idealized model, simulations with prolonged APD in the infarct BZ were carried out with the LV models to serve as a basis for comparison. In total, 140 simulations were performed to account for all combinations between reduced INa (or prolonged APD) and decreased *σ*_*m*_. Each simulation run took ∼13 h of execution time on 32 nodes of a high performance computer (SGI UV 1000).

#### VT induction due to prolonged APD in the BZ

Table 1 presents the number of LV models in which block/re-entry were detected (number of sustained re-entries is also shown in parenthesis). Conduction block was observed in four out of seven models with prolonged APD and preserved *σ*_*m*_ = 1.0. However, in only one model, the blocked beat degenerated into VT. Decreasing *σ*_*m*_ increased the probability of block in the BZ of the models in the cohort. However, re-entries only became more likely when *σ*_*m*_ was reduced to 10%. In this scenario, re-entry was detected in five LV models, three of which did not last for more than one cycle (nonsustained).

**Table 1:** **Vulnerability of the LV Model Cohort, n = 7, To Conduction Block and Re-entry as a Function of INa and***σ*_*m*_

*σ*_*m*_ (Scaling Factor)	Prolonged APD	INa (Scaling Factor)			
		0.10	0.25	0.50	1.00
1.00	4/1 (0)	7/3 (1)	4/0 (0)	1/0 (0)	0/0 (0)
0.50	5/0 (0)	7/3 (2)	4/1 (0)	2/0 (0)	0/0 (0)
0.25	6/1 (0)	7/5 (3)	7/2 (0)	3/0 (0)	1/0 (0)
0.10	7/5 (1)	7/5 (1)	7/3 (2)	7/1 (0)	6/0 (0)

Experiments with prolonged APD in the BZ are also included. Shown are the number of models in which block/re-entry (sustained re-entry) was detected.

**Figure 7:**
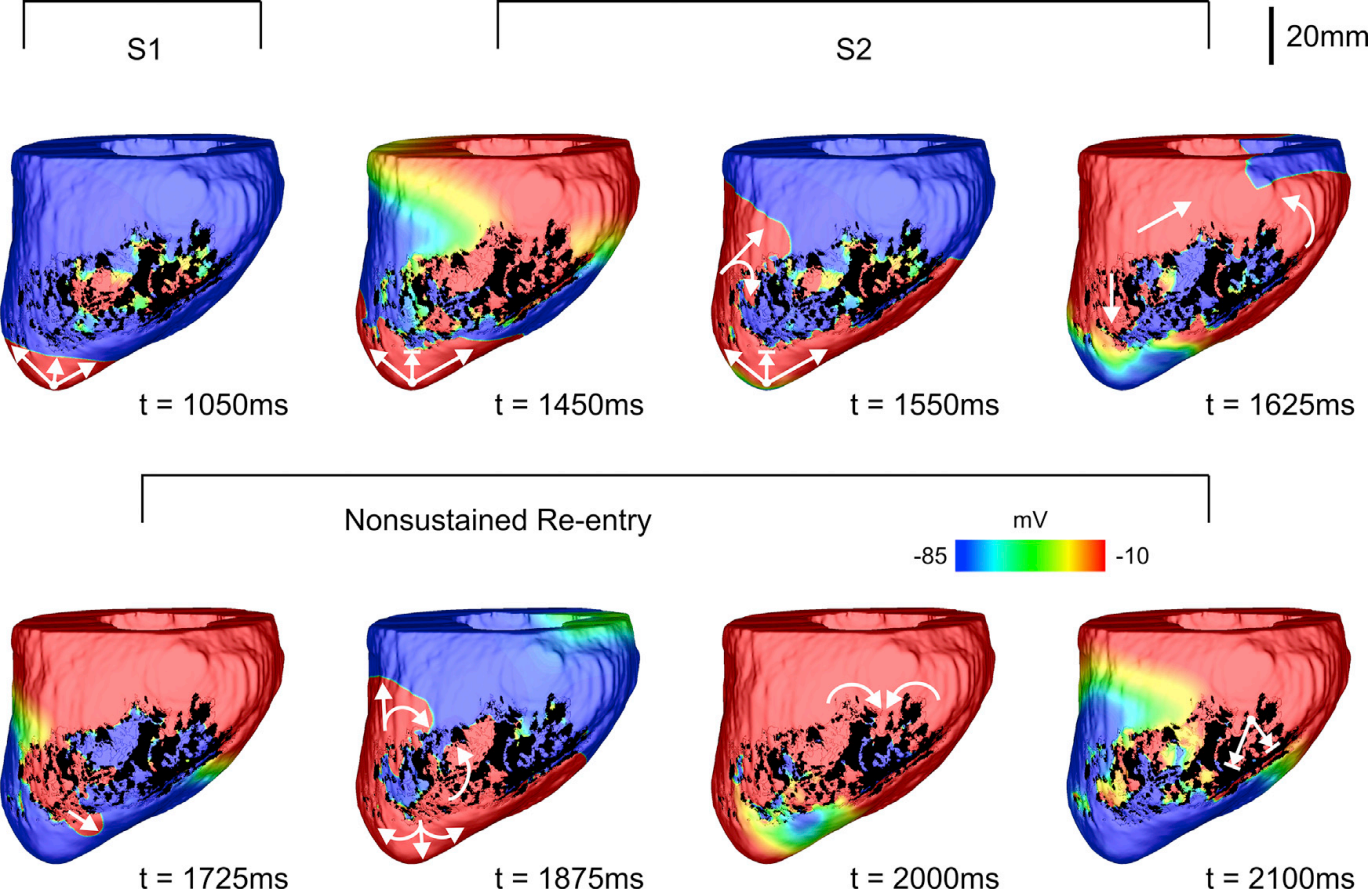
Nonsustained VT within LV model number 5 with prolonged APD and *σ*_*m*_ reduced to 10% in the infarct BZ. *V*_*m*_ maps at different times show VT induction following the S1–S2 pacing protocol. Arrows represent successful propagation. Lined arrows represent conduction block. VT induction in the LV model number 5 with prolonged APD in the BZ can be fully appreciated in Video S4. To see this figure in color, go online.

Fig. 7 shows *V*_*m*_ maps of the (last) S1 and S2 beats as well as the resulting nonsustained VT induced in LV model number 5 with prolonged APD and reduced *σ*_*m*_ (10% of its control value) in the BZ. At time t = 1050 ms, the last S1 beat propagates toward the infarct BZ. Note that there are sites inside the infarct that are still repolarizing from the previous S1 wavefront due to the prolonged APD assigned to the cells in the BZ. Like the 2D sheet with an idealized infarct in Fig. 3, the S2 wavefront initially blocks at the BZ (around t = 1450 ms) because the tissue there is still in the refractory period. The S2 travels around the BZ entering the infarct region from a distal site where tissue has regained excitability (see curved arrows at t = 1550 ms). The wavefront propagates slower inside the BZ toward the apex (t = 1625 ms), exiting to the myocardium at time t = 1725 ms. It can be seen that at t = 1875 ms, the re-entrant beat propagates throughout the LV as well as further into the infarct region. Finally, at around t = 2000 ms, all fragmented waves merge and enter the BZ from the base of the LV. The wavefront blocks at this region because the surrounding tissue is still refractory terminating the VT (t = 2100 ms), similar to Fig. 3 *A*. Conduction block and re-entry of the S2 wavefront in the LV model number 5 can be better appreciated in Video S4.

Video S4. Nonsustained VT Induction within the Image-Based LV Model Number 5 with Prolonged APD in the BZ See Fig. 7 for details. *V*_*m*_ of the last S1 beat (delivered at t = 1000 ms) followed by a premature S2 with a coupling interval of 320 ms that degenerated into a nonsustained re-entry (same scale as Fig. 7). RV septal (left) and LV endocardial cutaway (right) surfaces are shown.

#### VT induction due to reduced INa and structural remodeling in the BZ

The results of the analysis performed on the porcine cohort with different levels of reduced INa in the BZ are summarized in columns 3–6 of Table 1. Although conduction block was detected in LV models with only decreased *σ*_*m*_ in the BZ (last column of Table 1), VT was not inducible. Similar to the 2D experiments, normal INa led to normal propagation. Again, reduced INa alone was sufficient to provide the initial block for VT induction. However, re-entrant waves became more likely and more sustained when reductions in INa were combined with decreases in *σ*_*m*_.

**Figure 8:**
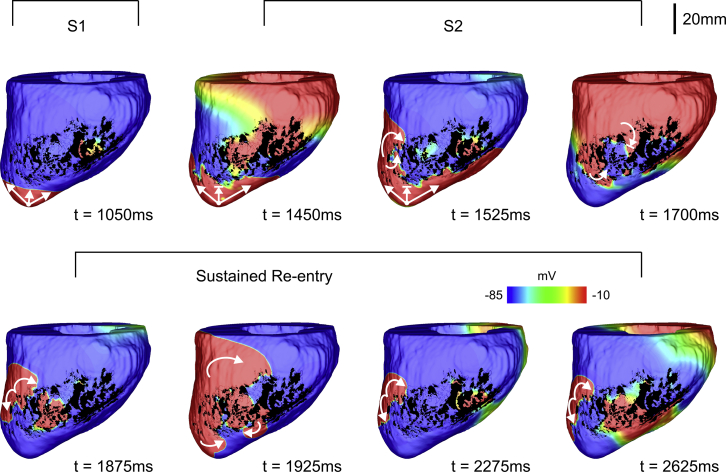
Sustained VT within LV model number 5 with reduced INa in the infarct BZ. *V*_*m*_ maps at different times showing VT induction following the S1–S2 pacing protocol. Arrows represent successful propagation. Lined arrows represent conduction block. VT induction in the LV model number 5 with reduced INa in the BZ can be fully appreciated in Video S5. To see this figure in color, go online.

Fig. 8 as well as Video S5 show VT induction in the same geometric LV model in Fig. 7 but with reduced INa (10% of its control value) in the BZ instead of prolonged APD. Similar to Fig. 7, the blocked S2 beat travels around the BZ, entering the infarct at a more distal region at t = 1525 ms as cells there have recovered excitability. Note that the wavefront travels slower inside the BZ (t = 1700 ms), leaving the infarct from a different region and at a later time (t = 1875 ms) when compared to the case with prolonged APD (t = 1725 ms). The re-entrant wavefront travels around the scar toward both the apex and base of the LV where it enters the BZ through small isthmuses (see Video S5 for more details). Unlike in the scenario illustrated in Fig. 7, the VT induced in LV model number 5 with reduced INa is sustained. The second and third cycles of the VT start around t = 2275 ms and t = 2625 ms, respectively, when the re-entrant wavefronts again leave the BZ to enter the myocardium.

Video S5. Sustained VT Induction within the Image-Based LV Model Number 5 with Reduced INa, 10% of Its Control Value, in the BZ See Fig. 8 for details. *V*_*m*_ of the last S1 beat (delivered at t = 1000 ms) followed by a premature S2 with a coupling interval of 320 ms that degenerated into a sustained re-entry (same scale as Fig. 8). Right ventricular septal (left) and LV endocardial cutaway (right) surfaces are shown.

Because of computational restrictions, the arrhythmogenic risk of structural remodeling as a result of fibrosis was investigated in only one LV model. Fibrotic amounts FIB of 25, 50, 75, and 90% were probed in the BZ of LV model number 5. Unlike in the 2D models, where 10 different patterns were generated, only one fibrotic texture for each FIB was created and added to the BZ. Conduction block was detected only in the case in which FIB corresponded to 90% of the BZ, but it did not degenerate into VT. Similar to the experiments in Figs. 5 and 6, fibrosis was combined first with reduced INa and then with decreased *σ*_*m*_. Although block was present, VT was not inducible in any of the experiments. VTs were detected only when fibrosis was combined with reductions in both INa and *σ*_*m*_. However, all re-entries in these experiments lasted only for one cycle.

## Discussion

The objective of this study was to determine whether factors associated with conduction slowing, rather than abnormal repolarization, can form a substrate for both arrhythmia induction and sustenance in the infarcted heart. To achieve this goal, we performed an in silico investigation on 2D idealized infarct tissue models as well as on a cohort of postinfarction porcine LV models constructed from ex vivo MRI scans. The electrophysiological properties of the infarct BZ were modified to represent different degrees of both functional and structural remodeling processes associated with slow CV. Functional remodeling was represented by reducing INa current density, whereas structural remodeling was accounted for by decreasing tissue conductivity and including fibrosis. The arrhythmogenic risk of these remodeling processes was compared to alterations promoting APD prolongation. Simulation results showed that the combination of reduced INa with either tissue conductivity or fibrosis could lead not only to conduction block but also to re-entry. Conduction block in the absence of APD prolongation was caused by reduced excitability due to reduced INa. Moreover, re-entries in experiments with both reduced excitability and tissue conduction were more likely to be sustained than experiments conducted with prolonged APD and decreased tissue conductivity as in the latter case, subsequent block often occurred because of a long-lasting repolarization.

### Arrhythmogenesis by prolonged APD

Experiments with isolated canine myocytes from the BZ 5 days after MI have shown that a decrease in repolarizing currents prolongs APD (10). However, recent experimental evidence has demonstrated that a shift in balance between inward and outward currents acts to shorten APD in the BZ while prolonging APD in remote zones (40). In contrast to the single-cell level, there is little compelling evidence for APD prolongation in the BZ of chronic infarct regions, particularly in the human ventricle (24). Many animal infarct models necessarily represent a much earlier healing phase of infarction, and the definition of the BZ regions (epicardial versus lateral (24)) are not necessarily consistent with the definition of the clinical BZ delineated from MRI images or in the catheterization laboratory. Despite these issues, in the human chronic infarcted BZ, repolarizing potassium channels may well still be downregulated as seen in the isolated myocyte experimental animal measurements (10). However, importantly, the effects of 3D well-coupled myocardium in these regions are thought to attenuate any local APD changes and subsequent gradients. Any localized change in APD will be modulated significantly by electrotonic coupling (41) as has been well shown for transmural APD gradients (42).

In the case of prolonged APD in the BZ, the mechanism of arrhythmogenesis via unidirectional conduction block is clear. A premature beat is blocked at the BZ region (which may remain refractory compared to neighboring healthy tissue due to the prolonged APD) being forced around the periphery of the scar. During this time, the BZ regions are recovering and later allowing penetration of the activation wave through diastolic channels that characterize the re-entrant circuit through the scar. Such basic mechanisms of arrhythmia induction have been clearly elucidated by anatomically detailed computational infarct models because of their inherent ability to dissect 3D activations and anatomy (12, 13, 14, 15, 16, 17, 18, 19, 20, 21, 22). This is the exact mechanism we also highlight in Figs. 3 *A* and 7 in this study. Were APD not to be prolonged at the tissue level, however, the question arises as to what mechanisms might precipitate unidirectional conduction block in the BZ region, tissue that is known to highly correlate with arrhythmic risk (2, 43).

### Arrhythmogenesis by reduced excitability and tissue conduction

Other electrophysiological and structural changes, in addition to the above-mentioned changes to repolarizing potassium currents, are also known to occur in the BZ regions, including downregulation of gap junctions (5), infiltration of (often patchy) fibrosis (6), reduced INa (44), and activation of fibroblasts (myofibroblasts) (45, 46). Individually, such changes all can have significant effects on the activation pattern and CV, with gap junction remodeling decreasing cell-to-cell conductivity (47), tortuous activation pathways enforced by patchy fibrosis causing significant macroscopic conduction delays (25, 26), and reduced INa slowing the action potential (AP) upstroke and cell-to-cell excitability, slowing CV (8).

As well as reducing CV, as we have demonstrated in this work, factors such as fibrosis density and reduced INa can also independently cause unidirectional block of premature stimuli within BZ regions (see Figs. 4, 5, and 6; Table 1). For reduced coupling intervals, sodium (Na^+^) channels are largely refractory and channel conductance is low; reduced Na^+^ channel density due to remodeling in the BZ further reduces the total INa current and can mean that BZ tissue remains refractory for conduction, whereas surrounding myocytes (with intact Na^+^ channel densities) have sufficient INa to become excited. This discrepancy underscores the mechanism for conduction block due to reduced INa in Figs. 3 *B* and 8. In the case of fibrosis, source-sink mismatches as wavefronts propagate around and through these complex fibrotic patterns have been shown to result in unidirectional block (27, 34, 48, 49). Microscopic tissue expansions, generated by fibrosis, effectively increase the local effective refractory period (12, 28, 29), meaning that conduction fails in fibrotic regions but propagates normally in surrounding healthy tissue, as highlighted in Fig. 3 *C*. Reducing tissue conductivity *σ*_*m*_ independently, however, was shown not to provide a substrate for unidirectional block (see Fig. 4). As suggested in previous works, reducing gap junction coupling between cells while slowing conduction also makes it safer and less prone to block (8, 50).

Finally, not considered in this work is the possible proarrhythmic substrate arising from the activation of fibroblasts in the wake of MI. Although the population of fibroblasts outnumber myocytes, they are electrophysiologically quiescent in the healthy heart (45). In the infarcted heart, fibroblasts are activated and differentiate into myofibroblasts in a healing response to MI to promote scar formation (46). Myofibroblasts can electrically couple to myocytes and may alter AP morphology and impulse conduction in a proarrhythmic way (51). Computer simulations on an infarcted rabbit model have shown that myofibroblasts reduce both the APD of remodeled BZ cells (with prolonged APD) and of healthy myocytes in surrounding tissue, below even levels of healthy controls (15). Dhanjal et al. (46) have demonstrated that slow CV is associated with the presence of myofibroblasts within the isthmus sustaining VT in the infarcted pig heart. A possible mechanism behind this conduction slowing was investigated previously in an in silico study (15). The authors showed that coupling between myofibroblasts and myocytes led to an elevation in resting membrane potential within the BZ (15). Elevated resting membrane potential causes a partial inactivation of Na^+^ channels, providing a similar mechanism of arrhythmogenesis in this region as modulated INa (by reducing channel maximal conductance) in Figs. 4 and 5.

### Arrhythmia sustenance

In the setting of scar-related VT, once the initial unidirectional block in the vicinity of the scar is established, the blocked wave propagates around/through the circuit setup by the scar and attempts to re-enter the site of initial block. Such re-entry is only successful if the tissue at this location has recovered sufficiently to be re-excited once more (13, 14). Critically, what this means is that for re-entry to be successful, the wavelength of propagation must be less than the physical pathlength established by the scar anatomy.

In this study, the anatomical pathlength in the 2D model was kept constant to assess the individual contributions of all parameters under investigation to the formation and sustenance of scar-related VTs. Any changes in the size of the scar/isthmus would directly affect the size of the re-entrant circuit and thus, VT, likelihood. On one hand, for smaller scars, the tissue within the isthmus would not have enough time to recover excitability (wavelength larger than the re-entrant circuit), leading to conduction block at the distal mouth (similar to the block of the re-entrant wave at t = 1800 ms in Fig. 3 *A*). Larger scars, on the other hand, would give rise to large anatomical pathways favoring re-entry formation (wavelength smaller than the re-entrant circuit). Similarly, the wavelength can be modulated by CV within the ventricles. *σ*_*ml*_ in the healthy myocardium was adjusted in all models to produce a longitudinal CV of 0.6 m/s (37). However, CV has been reported to range from 0.41 to 0.87 m/s (52, 53, 54, 55). Although overall CV increase is antiarrhythmic as it acts to increase wavelength, pathological conditions, such as heart failure, leading to slow conduction predispose the heart to arrhythmias (47). Structural and functional arrhythmogenic determinants were also assessed in silico in the context of atrial tachycardias (56). The authors showed that initiation and maintenance of re-entries depended critically on the size of the pulmonary veins as well as on heterogeneous and anisotropic conduction. Thus, slowed conduction through the scar is therefore well known to be arrhythmogenic (and is prerequisite of re-entry in this context), shortening, as it does, the functional re-entrant wavelength.

We show in this study that slowed conduction in the BZ, either due to reduced INa or decreased tissue coupling, provides this essential reduction in wavelength, making re-entry in this setting more likely (Figs. 4, 5, and 6; Table 1). The most substantial reduction comes in the combination of reduced INa and fibrosis (Fig. 5). This finding is corroborated by recent experimental work in a porcine model of chronic post-MI remodeling with increased afterload (57). The authors reported that a rise in interstitial fibrosis, under conditions of impaired excitability (rapid pacing and hypokalemia), promotes conduction-dependent arrhythmias. This is because any slight instabilities in source-sink mismatches brought about by the fibrosis are put under further stress by decreased INa, making slowing and block in this setting much more likely than the combination of reduced INa and decreased *σ*_*m*_ (Fig. 4). Decreased *σ*_*m*_ and fibrosis alone did not give rise to re-entries in any of the models. The combined effect of these structural remodeling processes, on the other hand, formed the substrate required for re-entry formation as shown in Fig. 6 (lower panel).

Although only one infarct representation was considered in the idealized 2D model, the number of LV models in the cohort (n = 7) allowed us to investigate different scar anatomies and re-entrant pathways in a robust way. We showed in the anatomically faithful porcine infarct models that reductions in INa alone could facilitate sustained re-entry, although re-entry was more likely when combined with reductions in *σ*_*m*_ (see Table 1). This is again due to reduced INa enhancing source-sink mismatches caused by structural heterogeneities found in the BZ of the LV models. Although the combination of reduced INa and fibrosis led to re-entry in the idealized 2D model, this was not the case when fibrosis was added to the LV model number 5. The ex vivo imaging data used to build the LV models contained high-detailed information about the structural heterogeneities in the infarct region (see Fig. 2). The synthetic fibrotic patterns added to the BZ may have altered these heterogeneities that sustained VT in the nonfibrotic model.

An important finding of this work was that representing prolonged APD in the BZ was, on the whole, not able to induce sustained re-entry in the porcine cohort (Fig. 7; Table 1), despite being able to induce the initial unidirectional block. Our explanation for this phenomenon is that the increased APD in the BZ lengthens wavelength beyond a level that is sustainable by the re-entrant circuit represented by the particular anatomical scar substrate. However, in chronic MI patients, the anatomical re-entrant pathways may be longer, allowing the healthy myocardium at both exits of the isthmus to repolarize and thus forming a circuit capable of sustaining re-entry. Also, if conduction in the infarct is substantially impaired, the tissue within the isthmus/BZ may recover excitability even if the APD in this region is prolonged. In fact, only when tissue conductivity was severely decreased (10% in Table 1) was sustained re-entry seen in one LV model because of the concordant reduction in wavelength through reduced CV around the circuit. The antiarrhythmic nature of prolonged APD is also underscored in relation to the use of certain antiarrhythmic drugs, such as amiodarone, whose mechanism of action is thought to be in the prolongation of phase 3 of the AP (58).

Here, we did not include prolonged APD along with reduced INa and other ionic changes (changes in L-Type calcium, for example) as has been implemented in more complete representations of the remodeled BZ in other studies (12, 13, 15, 16, 17, 18, 19, 20, 21, 22) as our goal was to independently dissect the effects of individual remodeling processes. In such other work, more sustained episodes of induced arrhythmias were indeed witnessed. Potentially, this suggests that the decrease in CV by the additional reduction of INa (typically reduced to 38% in these other studies) is the critical factor in sustaining arrhythmias. Alternatively, it could also suggest that the balance between the specific functional wavelength relative to the anatomical (scar) re-entrant pathlength is more delicate in some species than others (i.e., once induced, particular species are more robust to slight differences in APD around the re-entrant circuit). This may also suggest why previous simulation work in human (18, 21, 22) and rabbit (13, 15, 17) chronic infarct models that have imposed similar functional remodeling (including prolonged APD) have not reported issues in simulating sustained re-entries. However, it is also interesting to note that a very recent work examining the re-entrant dynamics in similar ex vivo MRI-derived porcine models enforced a prolonged APD (and reduced CV) within BZ tissue only for arrhythmia induction (following the S1 beat); once induced, this was removed to facilitate arrhythmia sustenance, presumably due to issues in sustaining arrhythmias with such prolonged APDs (59).

### Large-scale computational representation of BZ remodeling

Facilitating the practical deployment of large-scale computational image-based models within a clinical environment requires maximizing the simplification of modeling strategies, primarily to limit model complexity and the computational load of these large, anatomically detailed human models. Although in the past decade, the use of high-performance technologies, such as graphic processing units, have become a promising alternative to reduce the need for modeling simplification (60, 61, 62), the numerical solution of modern models remains computationally vastly demanding. For example, representing the intricate patterns of patchy fibrosis within BZ regions requires a model mesh resolution (in the BZ, at least) of the order of the typical dimensions of microscopic fibrotic bundles (shown to be a few hundred microns (25)). Furthermore, faithful representation of the effective presence of myofibroblasts within the myocardium may also necessitate further refinement to levels of the order of 50 *μ*m (15). However, the majority of models previously used for patient-specific modeling have typically had ventricular mesh refinement levels at 350 *μ*m (21, 22). Necessitating such fine resolutions may increase model sizes to computationally prohibitive levels for use as a clinical tool.

Changing INa, mechanistically, impacts tissue excitability causing unidirectional block under rapid pacing, and has the effect of slowing conduction. Similarly, myofibroblast coupling can reduce tissue excitability, while patchy fibrosis provides the substrate for slow conduction in infarcted tissue (as highlighted in Fig. 3, *B* and *C*). Thus, reducing INa below the reported 38% reductions from experimental isolated myocyte preparation levels (44) (as done in many of the simulations in this work) may provide a computationally efficient way in which to represent the combined effects of multiple remodeling mechanisms (reduced INa itself, patchy fibrosis, myofibroblasts), removing the need for additional mesh refinement in the BZ.

### Clinical relevance

A detailed understanding of the mechanisms underlying the initial block that leads to re-entry within the infarcted heart is necessary for the successful development of therapeutic strategies for scar-related VTs. Conduction block has been often associated with electrophysiological changes that prolong APD in cells within the BZ (10). However, recent experimental evidence has shown that APD is shorter in isolated cells from the BZ of failing hearts (40). Moreover, tissue (un)coupling might attenuate or exacerbate APD gradients (42). Nevertheless, accurate measurement/assessment of the electrophysiological remodeling in in vivo experimental and clinical settings is challenging.

Models and simulation can help interpret information at multiple scales. Computational modeling is an ideal platform for investigating arrhythmic mechanisms ranging from subcellular to the whole organ. The in silico experiments in this study allowed us to thoroughly assess the role played by different functional and structural remodeling processes into the formation and sustenance of VTs after MI. In-depth knowledge of functional remodeling processes can pave the road to new pharmacological therapies aiming to reduce VT frequency, decrease implantable cardioverter-defibrillator shocks, and improve the efficacy of antitachycardia pacing therapies. Similarly, elucidation of the structural substrate could be used alongside gene and tissue engineering treatments to ameliorate cardiac performance, reducing the risk of hospitalizations and improving the quality of life. Furthermore, recent clinical studies very successfully showed that computational models can outperform traditional approaches in both predicting arrhythmogenic risk associated with MI (21) and identifying optimal targets for catheter ablation of VTs (22). Accurate parameterization of these models can further improve their potential as a tool for noninvasive investigation and treatment of VTs.

### Limitations

In this study, a human and not a porcine model was used to simulate the AP in all tissue models. To the best of our knowledge, there is currently no model describing the ionic membrane dynamics in porcine ventricular myocytes. Nevertheless, APD in isolated cells from the pig heart is similar to human ventricular myocytes (63). Furthermore, a homogeneous APD distribution was used in the LV models. Although heterogeneities in APD within the ventricular wall might alter VT dynamics, they would not change the basic phenomenon of conduction block in the BZ. Moreover, fibrosis was represented in the geometrical models by randomly transforming myocytes into nonconducting tissue. Although more sophisticated approaches exist (64), the overall activation behavior would be qualitatively similar because the wavefront would still have to travel through tortuous pathways. Finally, as discussed above, the combination of reduced INa and fibrosis did not result in VT in the LV model. In addition to the possible disruption in structural heterogeneities in the infarct region by the addition of synthetic fibrosis, only one fibrotic pattern was assigned to the BZ of LV model number 5 because of computational restrictions. Other fibrotic patterns may be more arrhythmogenesis than the one we created.

## Conclusions

In this study, we have employed computational models with different complexities to investigate whether functional and structural remodeling promoting conduction slowing can lead to arrhythmia. This has not been robustly investigated in the literature so far and may have critical implications in the use of computational models to assess patient-specific arrhythmogenic risk and improve therapy planning. Our in silico experiments demonstrated that the downregulation of INa not only slows CV but also hinders tissue excitability, providing a potent substrate for conduction block in the absence of abnormal repolarization. When combined with decreased tissue coupling (downregulation of gap junctions or fibrosis), reduced INa further shortened the wavelength of the cardiac propagation, which resulted in more sustained re-entries. We conclude that a combination of functional and structural conditions associated with slow conduction provides a substrate for both the initiation and maintenance of scar-related VTs. This represents a new finding as it demonstrates for the first time, to our knowledge, that functional and structural alterations associated with slow CV rather than prolonged APD, often implemented in current computational models as a substrate for initial block, can lead to sustained VTs in the chronically infarcted heart.

## Author Contributions

Designed study, F.O.C. and M.J.B.; Performed simulations, F.O.C.; Performed experiments, J.W., R.N., S.R., and M.O’N.; Analyzed data, F.O.C., J.W., and M.J.B.; Drafted manuscript, F.O.C., J.W., R.N., S.R., M.O’N., G.P., and M.J.B.
